# Efficacy of motor imagery in post-stroke rehabilitation: a systematic review

**DOI:** 10.1186/1743-0003-5-8

**Published:** 2008-03-14

**Authors:** Andrea Zimmermann-Schlatter, Corina Schuster, Milo A Puhan, Ewa Siekierka, Johann Steurer

**Affiliations:** 1 Zurich University of Applied Sciences, Winterthur, Switzerland; 2 Reha Rheinfelden, Salinenstrasse 98, 4310 Rheinfelden, Switzerland; 3 Oxford Brookes University, Oxford, United Kingdom; 4 Horten Centre for patient-oriented research and knowledge transfer, University of Zurich, Switzerland; 5 Department of Neurology, University Hospital Zurich, Switzerland

## Abstract

**Background:**

Evaluation of how Motor Imagery and conventional therapy (physiotherapy or occupational therapy) compare to conventional therapy only in their effects on clinically relevant outcomes during rehabilitation of persons with stroke.

**Design:**

Systematic review of the literature

**Methods:**

We conducted an electronic database search in seven databases in August 2005 and also hand-searched the bibliographies of studies that we selected for the review.

Two reviewers independently screened and selected all randomized controlled trials that compare the effects of conventional therapy plus Motor Imagery to those of only conventional therapy on stroke patients.

The outcome measurements were: Fugl-Meyer Stroke Assessment upper extremity score (66 points) and Action Research Arm Test upper extremity score (57 points).

Due to the high variability in the outcomes, we could not pool the data statistically.

**Results:**

We identified four randomized controlled trials from Asia and North America. The quality of the included studies was poor to moderate. Two different Motor imagery techniques were used (three studies used audiotapes and one study had occupational therapists apply the intervention). Two studies found significant effects of Motor Imagery in the Fugl-Meyer Stroke Assessment: Differences between groups amounted to 11.0 (1.0 to 21.0) and 3.2 (-4 to 10.3) respectively and in the Action Research Arm Test 6.1 (-6.2 to 18.4) and 15.8 (0.5 to 31.0) respectively. One study did not find a significant effect in the Fugl-Meyer Stroke Assessment and Color trail Test (p = 0.28) but in the task-related outcomes (p > 0.001).

**Conclusion:**

Current evidence suggests that Motor imagery provides additional benefits to conventional physiotherapy or occupational therapy. However, larger and methodologically sounder studies should be conducted to assess the benefits of Motor imagery.

## Background

Annually 15 million people worldwide suffer from a stroke. Of these, five million remain permanently disabled, despite intensive rehabilitation programs, and are no longer capable to care for themselves [1].

During the first few days following the incident, lifesaving and thrombolytic therapies have priority. However, as soon as possible [2,3], patients should exercise to activate the process of recovery and neural re-organization [4-6].

Different rehabilitative approaches are used for post-stroke treatment. One of them is Motor Imagery (MI). MI was initially developed to improve the performance of athletes [7-9] and has been adopted in rehabilitation programs for persons with stroke [10] to support motor recovery [11,12].

Mental imagery refers to the active process by which humans experience sensations with or without external stimuli [13]. It is an active process during which a specific action is reproduced within working memory without any real movements [13,14]. Studies [15,16] demonstrate that during MI sessions partially the same brain areas are as activated as during functional tasks.

Function, behavior, and performance are rehearsed mentally as if the person is actually performing them [17]. From sports literature it is well known that MI, when applied in addition to functional training, is more effective than MI or functional training alone [18]. However, Sharma [12] has pointed out that MI training alone produces less improvement than functional training.

An advantage of MI is that patients can practice it independently during the regeneration phase between two physical therapy sessions. MI can also be practiced in all stages of stroke recovery [13]. In an early stage of recovery, MI allows patients to mentally practice a task which they cannot yet carry out physically due to motor impairment. However, it has not been determined yet, when it is best to start with MI.

Although there is sufficient evidence that MI can improve function in healthy subjects [13], only a few, small randomized controlled trials have evaluated the effect of MI in stroke patients. To explore the potential role of MI in post-stroke rehabilitation and to outline a potential research agenda, we conducted a systematic review of all randomized controlled trials that analyze the effect of MI on patients after a cortical stroke.

## Methods

### Identifications of studies

We searched the following databases for relevant studies: Ovid MEDLINE (Ovid version, from inception to August 2005), PEDRO (online version, University of Sydney, Australia, August 2005), PsycINFO (from 1967 to July 2005), Psyndexplus (from 1977 to June 2005), CINAHL (Cumulative Index to Nursing & Allied Health Literature, from 1982 to July 2005), Cochrane Central Register of Controlled Trials (Oxford, UK, 2004, issue 1), and Scopus (from inception to August 2005).

The detailed search strategy for the MEDLINE search is described in the appendix.

The search was conducted without restrictions to language or year of publication.

We also hand-searched the bibliography of all studies ordered in full text.

### Selection criteria

We included all randomized controlled trials that compare conventional physiotherapy or occupational therapy to MI combined with conventional physiotherapy or occupational therapy in post-stroke rehabilitation. We excluded mental practice based on computer-animated techniques, because these techniques are not available in most rehabilitation settings. Only studies about patients with a first episode of stroke were considered with no restrictions concerning age or time since onset of stroke.

The outcome assessment had to be clinically and functionally relevant, for example performance of specific tasks and activities or health-related quality of life.

### Study selection

After the electronic database search, the two reviewers (AZ and CS) screened the titles and the abstracts of all resulting references (N = 2116) independently. They recorded their decision about in- or exclusion in an EndNote (Thomson Wintertree Software Inc) file. In cases where reading the abstracts was not enough to determine whether or not to include a study, the entire study was ordered. The reviewers then evaluated the retrieved full-text studies and made a decision on inclusion or exclusion according to the criteria specified above.

The reviewers also hand-searched the bibliographies of the full-text studies and reviews to identify further relevant studies. Each reviewer's decisions as well as the final decisions on journal articles were recorded in the EndNote file. Studies that did not fulfill all of the predefined criteria were excluded and their bibliographic details were listed with the specific reason for their exclusion.

### Data extraction

The reviewers independently recorded details about study design, interventions, outcome measurement methods, and results in a predefined form. Both also separately evaluated the quality of the included trials based on a detailed list of quality items (see table 1). A third reviewer (JS) resolved any discrepancies when the two reviewers disagreed. We tried to contact the authors of the selected studies for further information about missing data but did not get any response.

**Table 1:** Quality assessment of the included studies

	Liu [22]	Page [24]	Page [25]	Page [23]
Selection of prognostic homogenous study population (disease progression)	1	3	3	3
Concealment of random allocation	1	3	3	3
Prestratification of prognostically relevant variables	3	3	3	3
Random allocation (description of procedure)	2	1	1	3
Registration of loss to follow-up	1	3	4	4
Blinding of patients	4	4	4	4
Blinding of persons who implement interventions	4	4	4	4
Registration of co-interventions that bear on outcome for each group	3	3	1	3
Blinding of persons who assess treatment effects	3	1	1	3
Check to what extent blinding was successful	3	3	3	3

1 = Item is properly addressed, 2 = Item is partially addressed, 3 = Item is not properly addressed or not stated, 4 = Item is not applicable

### Quality assessment

The two reviewers appraised all included trials based on a pre-defined list of selected quality items assessing components of internal validity [19] (Table 1). In case of any discrepancy, we obtained the opinion of a third reviewer. We divided all quality items into the following four categories: 1 = item is properly addressed; 2 = item is partially addressed (authors mentioned that this quality item was fulfilled but did not describe the procedure); 3 = item is not properly addressed or not stated (the item was not fulfilled or the authors did not mention it); 4 = item is not applicable.

### Analysis

We summarized the results of the data extraction and the quality assessments in structured tables. This compilation allowed us to examine the variation in patient characteristics, study quality and results.

Because of the heterogeneity in the studies we could not perform a data pooling for a meta-analysis. Wherever possible, we presented point estimates and 95% confidence intervals of single study results. Since not all results were presented with a confidence interval of 95%, we used the standard deviation from one study [20] to estimate the confidence interval of the other studies [21].

## Results

### Study selection

**Figure 1:**
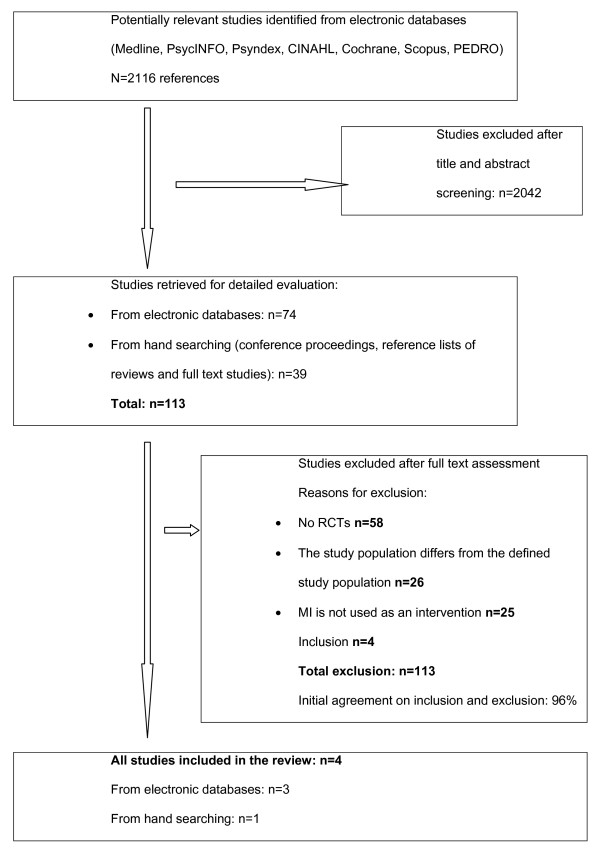
Flow diagram of study selection process.

Figure 1 shows the study selection process and the reviewers' agreement on study inclusion. Our search yielded 2116 potentially relevant citations after removing duplicates. 113 articles were selected for closer evaluation. Of these, we included four RCTs [22-25]. Reasons for the exclusion of the other 109 studies were: no RCTs (n = 58), study population differed from the pre-defined study population (n = 26), MI was not used as an intervention (n = 25). The two reviewers agreed in 96% of the cases on inclusion or exclusion of the studies.

### Characteristics of the included studies

(Table 2 provides descriptive data for the included studies)

**Table 2:** Characteristics of included randomized controlled trials

**Study**	**Number of patients**	**Gender (% male)**	**Mean age in years (± SD if available) range (if available)**	**Time since stroke (months)**	**Intervention**	**Outcomes**
Liu [22]	46 with a first unilateral cerebral infarction	48	MI group: 71.0 (± 6.0) Controls: 72.7 (± 9.4)	0.5	**Intervention group:** 60 minutes PT sessions five days a week for 3 weeks. Plus: motor imagery five 60 minutes sessions per week for 3 weeks. OT's provided the motor imagery training.	FMSA CTT2 Task performance test
					**Controls:** 60 minutes PT sessions five days a week for 3 weeks. Plus instead of imagery: a demonstration-then-practice method for the same tasks as in the MI group for five 60 minutes sessions per week for 3 weeks. OT's provided the demonstration than practice training.	

Page [24]	13 with a unilateral cerebral infarction	77	64.6 range:54–79	6.5	**Intervention group:**conventional therapy (OT and PT) 3 times/week, in 60 minutes segments for 6 weeks. Plus: 10 minutes audiotape with cognitive visual images + using such a tape at home twice a week.	FMSA ARAT
					**Controls:** Conventional therapy (OT and PT) 3 times/week, in 60 minutes segments for 6 weeks. Plus instead of imagery: 10-minutes tape containing stroke information + using such a tape at home twice a week.	

**Study**	**Number of patients**	**Gender (% male)**	**Mean age in years (± SD if available)**	**Time since stroke (months)**	**Intervention**	**Outcomes**

Page [23]	11 with a stroke	82	62.3 (± 5.1) range: 53–71	24	**Intervention group:** A set of ADL's is practiced through PT 2 times/week for 30 minutes for 6 weeks. Plus after PT participants received 30 minutes MP intervention. And they also practice it mentally at home.	ARAT MAL
					**Controls:** A set of ADL's is practiced through PT 2 times/week for 30 minutes for 6 weeks. Plus instead of MP: after the PT session they received a session of relaxation techniques for 30 minutes.	

Page [25]	16 with a unilateral cerebral infarction	100	63.2 (± 4)	22	**Intervention group: **OT: 3 times/week in 30 minutes sessions for 4 weeks. Plus: an imagery intervention lasting 20 minutes after the OT session.	FMSA
					**Controls: **OT: 3 times/week in 30 minutes sessions for 4 weeks. Plus instead of MP: after OT session a 20 minutes tape with instructions and information requiring the patients' attention and participation and on the causes and the pathology of strokes.	

Time elapsed since stroke ranged from a few days (mean: 12.3 days) to several years (mean: 23.8 months). Three studies [23-25] were carried out in North-America and one in Asia [22]. The study populations were quite homogeneous in terms of age but heterogeneous in aspects such as gender, dominant limb, affected side, and time elapsed since the incident (table 2).

Only one study [24] assessed the individual's ability to imagine using the Movement Imagery Questionnaire (MIQ) [26].

Duration and frequency of MI interventions varied between ten minutes [24] and one hour a day [22] with three to five sessions per week. The shortest intervention period lasted three weeks [22], the longest six weeks [23,24].

All studies compared MI plus conventional physiotherapy or occupational therapy to only conventional physiotherapy or occupational therapy. None of the included studies analyzed the effect of MI alone.

One study [22] trained the patients to carry out specific tasks using the technique of MI. In the first week, the primary objective was task analysis enhancement: Patients had to identify each step of the task with the help of MI and picture cards showing the task. In the second week, the primary objective was problem identification: patients had to visualize their own performance and identify the problems encountered and the solutions in each task step by means of MI. The third week focused on task performance: Patients had to imagine performing the task and then carry it out. In this study occupational therapists applied MI. As control intervention Liu et al. used a so-called "demonstration than practice method" (an occupational therapist demonstrated the same tasks as used in the MI group, afterwards patients had to practice this demonstrated tasks.

In the studies published by Page [23-25], patients had to listen to an audiotape with an introduction on relaxation, some suggestions for external, cognitive visual images, and instructions to refocus into the room. Duration of the tapes varied from ten [24] to 30 minutes [23].

All studies published by Page used conventional therapy plus information about strokes [24,25] or relaxation techniques [23] whereas Liu et al. [22] used a session with demonstration than practice method of the trained task as a control intervention.

None of the included studies evaluated the patients' satisfaction with the intervention.

### Quality assessment

The quality of the studies was moderate to poor. Table 1 summarizes the methodological quality of all the included studies.

None of the included studies used pre-stratification.

Two items (blinding of persons who implemented the interventions and blinding of patients) were not applicable in any studies. We applied the quality assessment in a restrictive manner and considered audiotapes with information about strokes and relaxation techniques as not being a "blinding procedure".

The registration of any co-intervention(s) was properly addressed in one study [23], yet three studies did not address this issue [22-25].

Blinding of the assessors was properly addressed in two studies [23,24] but not at all addressed in the others [22,25].

### Effects of motor imagery

In three studies the outcomes were measured with Fugl-Meyer Stroke Assessment (FMSA) and in two studies with the Action Research Arm Test (ARAT). The ARAT is an assessment for measuring specific changes in function of the upper extremity (grasp, grip, pinch and gross movements) for persons with hemiplegia. The test has a total score of 57 points [27]. In addition the FMSA upper extremity subscale is an assessment for movement, reflexes, coordination and speed with a total score of 66 points. For the ARAT the minimal clinically important difference is estimated to be 5.7 [28] whereas for the FMA the minimal clinically important difference is not estimated yet, but Gladstone proposes a 10% change of total score to be relevant.

**Figure 2:**
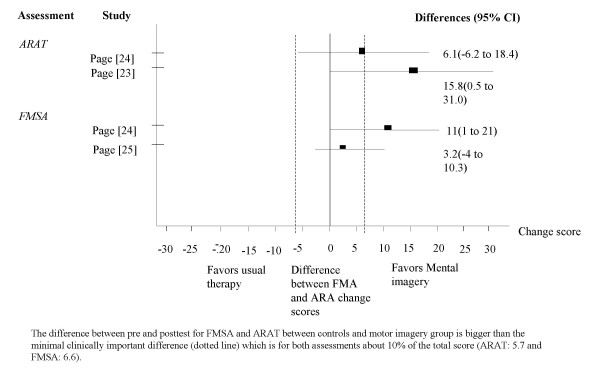
Differences between ARAT and FMSA upper extremity change scores.

Table 3 summarizes the results of the included studies and figure 2 shows the forest plots of the ARAT and the FMSA. We draw a dotted line to point out the minimal clinically important difference of ARAT and FMSA upper extremity score.

**Table 3:** Effects of MI

**Study**	**Assessment**	**Time of measurement**	**Results**
**Liu [22]**	FMSA upper extremity subscales, CTT	Pretest, Posttest after the inter-vention, follow-up after one month	Not significant
	Trained Tasks, set 1		Not significant
	Trained tasks, set 2		significant
	Trained tasks, set 3		significant
	Untrained tasks		significant
	Trained tasks, set 3, follow up		significant
**Page [25]**	FMSA, upper extremity subscales	Two pretests within one week, one posttest after the intervention	% Improvement MI group: 35.98 (10.17) Controls: 21.15 (4.87) No significance level is reported in this study.
**Page [24]**	FMSA, upper extremity subscales	Two pretest within one week, one posttest after the intervention	Improvement: MI group: 13.8 Controls: 2.9 No significance level is reported in this study.
	ARAT		Improvement: MI group: 16.4 Controls: 0.7 No significance level is reported in this level.
**Page [23]**	ARAT	Two pretests within one week, one posttest after the intervention	significant
	Motor Activity Log Amount of Use (AOU)		Improvement: MI group: 1.6 Controls: 0.4 No significance level is reported in this study.
	Motor Activity Log Quality of Movement (QOM)		MI group: 2.2 Controls: 0.2 No significance level is reported in this study.

Liu [22] found no significant difference between the FMSA upper extremity subscale and the Color Trail Test (CTT), but did find a significantly higher level of performance in the trained as well as untrained tasks for the imagery group. The trained tasks in week three were also evaluated in a one-month follow-up and the difference between the two groups was considered significant for the intervention group. Since Liu et al. [22] only reported that the FMSA was not significant. We were not able to display their results in figure 2. Page [24] reported substantial increases in the FMSA upper extremity subscales and the ARAT scores for the intervention group. The difference between the two groups exceeds the clinically important difference [28-30].

Page [23] detected a significant change in the ARAT score for the intervention group and remarkable changes concerning the Amount of Use (AOU) and the Quality of Movement (QOM) of the Motor Activity Log (MAL) [31].

Page [25] found a 35.98% (± 10.17%) improvement in the FMSA upper extremity subscale for the intervention group compared to 21.15% (± 4.87%) for the control group, but no significance levels were reported in this study.

## Discussion

Our systematic review indicates that there is modest evidence supporting the additional benefit of MI compared to only conventional physiotherapy in patients with stroke. Three studies [23-25] proved the positive effects of MI interventions on the ARAT and the FMSA and one study [22] stated significant effects on task-related outcomes, but not on the ARAT and the FMSA. Two studies [23,24] found higher mean change scores than the minimal clinically relevant difference in the ARAT and in the FMSA respectively.

The methodological quality of included randomized controlled trials with small sample size (n = 11 – 46) limits the findings of this review. The results of this review are only valuable for short-term effects of MI on functional outcomes. The presentation of data in the analyzed studies (for example: p-values of differences between the groups) complicated the data extraction and further analysis. This review cannot answer questions concerning the best time for an MI intervention because of the variability of time elapsed since the stroke event in the different patient samples. For the same reason, this review can also not respond to questions concerning the optimal duration or frequency of the intervention or the fatigue appearance in stroke patients. Since none of the included studies assessed how patients coped with the treatment, this review cannot draw any conclusions about the effect of the patients' motivation on the efficacy of MI. The authors of this review are not aware of any study which assesses whether a patient's ability to take part in the decision-making process influences the effectiveness of MI.

Although evidence exists that patients should start exercising as soon as possible [2,3], Byl et al. [32] found evidence that individuals > 6 months after stroke can achieve high levels of function following directed practice based on the principles of neuroplasticity. Since these results are based on functional exercises, it is unclear if they can be adapted to MI. From studies with athletes it is well known that it is an advantage for motor learning if the athlete is familiar with MI techniques. Isaac et al. [33] noted that subjects with a specialization such as elite athletes, air traffic controllers or pilots achieve significantly better results in vivid imagery than matched controls.

In June 2006, a systematic review [34] on the same topic also included one Controlled Clinical Trial (CCT) [35], two patient series [36,37], and three single case reports [38-40]. The results of theses studies support the results found in the four RCTs. Braun et al. [34] applied different quality assessment criteria and judged the quality of the included studies moderate to sufficient. They found "some evidence that mental practice as an additional therapy has effects on recovery after a stroke" but also stated that "mental practice and the outcome measurement are not standardized and thus difficult to compare." They advise further research based on a clear definition of the content of mental practice using standardized measurement methods. In contrast to Braun et al. [34] we presented data of three studies in a quantitative manner with forest plots of the ARAT and the FMSA to facilitate the interpretation of the effects of MI.

For further research, the authors recommend studies of better methodological quality, bigger sample size, and longer follow-up. Further research is also necessary to determine the optimum time for the intervention and the duration of the intervention, and to analyze the influence of motivation on the efficacy of MI.

MI appears to be an attractive treatment opinion, easy to learn and to apply and the intervention is neither physically exhausting nor harmful. Therefore, the authors believe that MI may generate additional benefit for patients.

## Competing interests

The author(s) declare that they have no competing interests.

## Authors' contributions

AZ participated in the study design, the study selection process the data extraction, performed the data analysis, and drafted the manuscript. CS participated in the study selection process, the data extraction and revised the manuscript. MP participated in the study design, the data analysis and revised the manuscript. ES revised the manuscript. JS participated in the study design and revised the manuscript. All authors read and approved the final manuscript.

## Appendix

We used the following search terms for MEDLINE, PsycINFO, Psyndex, Cochrane, CINAHL, Scopus, PEDRO

1 imagery.mp,hw. (953)

2 (imaginat$ or imagine$).mp,hw. (784)

3 (mental adj (practice or preparation or rehearsal or therapy)). mp,hw. (2006)

4 biofeedback$.mp,hw. (1023)

5 motor learning.mp,hw. (294)

6 neuronal plasticity.mp,hw. (254)

7 nerve cell plasticity.mp,hw. (2)

8 stroke?.mp,hw. (9395)

9 hemipare$.mp,hw. (493)

10 hemiple$.mp,hw. (1295)

11 apople$.mp,hw. (20)

12 cerebrovascular disorder$.mp,hw. (597)

13 exp "intracranial embolism and thrombosis"/(95)

14 exp intracranial hemorrhages/(1212)

15 exp carotid artery disease/(427)

16 exp cerebral ischemia/(791)

17 exp cerebral vascular accident/(9144)

18 exp brain ischemia

19 exp basal ganglia cerebrovascular disease

20 exp cerebral hemorrhage

21 exp cerebral ischemia

22 exp cerebrovascular accidents

23 exp paralysis/(3528)

24 exp paresis

25 or/1–7 (5113)

26 or/8–18 (16704)

27 19 and 20 (265)
